# Immunomodulatory Role of Mesenchymal Stem Cell Therapy in Vascularized Composite Allotransplantation

**DOI:** 10.1155/2016/6951693

**Published:** 2016-10-16

**Authors:** Richard Heyes, Andrew Iarocci, Yourka Tchoukalova, David G. Lott

**Affiliations:** ^1^Department of Otorhinolaryngology-Head and Neck Surgery, Mayo Clinic, Phoenix, AZ, USA; ^2^Head and Neck Regeneration Program, Mayo Clinic Center for Regenerative Medicine, Phoenix, AZ, USA

## Abstract

This review aims to summarize contemporary evidence of the in vitro and in vivo immunomodulatory effects of mesenchymal stem cells (MSCs) in promoting vascularized composite allotransplant (VCA) tolerance. An extensive literature review was performed to identify pertinent articles of merit. Prospective preclinical trials in mammal subjects receiving VCA (or skin allograft) with administration of MSCs were reviewed. Prospective clinical trials with intravascular delivery of MSCs in human populations undergoing solid organ transplant were also identified and reviewed. Sixteen preclinical studies are included. Eleven studies compared MSC monotherapy to no therapy; of these, ten reported improved graft survival, which was statistically significantly prolonged in eight. Eight studies analyzed allograft survival with MSC therapy as an adjunct to proven immunosuppressive regimens. In these studies, daily immunosuppression was transiently delivered and then stopped. In all studies, treatment-free graft survival was statistically significantly prolonged in animals that received MSC therapy. MSCs have been safely administered clinically and their use in renal transplant clinical trials provides evidence that they improve allograft transplant tolerance in clinical practice. There is potential for MSC induction therapy to overcome many of the obstacles to widespread VCA in clinical practice. Preclinical studies are needed before MSC-induced VCA tolerance becomes a clinical reality.

## 1. Introduction

Vascularized composite allotransplants (VCA), consisting of heterogeneous tissues from different germ layers including skin, muscle, bone, fat, nerves, and lymph nodes, significantly differ from other solid organ transplants in their extreme and diverse antigenicity requiring potent immunosuppressive regimens. In addition, VCA tend to be life-enhancing (as opposed to the life-saving nature of the solid organ transplantation) where success is perceived by functional outcomes and subjective recipient satisfaction. To date, over 100 upper limb, around 40 facial, and a handful of laryngeal VCA have been performed. Although functional outcomes have largely surpassed early expectations, serious complications and chronic rejection have been reported [1–3].

Precise immunosuppressive regimens vary from center to center, but triple therapy with tacrolimus, mycophenolate (MMF), and prednisone has been universally utilized in facial transplants [1]. These immunosuppressive doses are high, implying a substantial risk of toxicity with infectious and neoplastic consequences; and yet acute episodes of rejection have been reported in almost all facial transplant recipients [1]. In head and neck VCA, the transfer of mucosal surfaces, each with their unique floral composition, adds to the risk of donor-derived infections [4]. Although they are usually mild and can be medically managed ensuring graft survival [1, 5], one case associated with deadly posttransplant infections has also been reported [6]. Due to this increased risk of donor-derived bacterial, viral, and fungal infections, prophylactic antibiotic therapy is obligatory.

The relative risk of malignancy may be 400 times greater in those on lifelong immunosuppression compared to the general population, and neoplastic complications have been experienced in VCA [7]. An Epstein-Barr virus-related posttransplant B-cell lymphoma has been reported in facial transplant patients [3]. On removal of the first laryngeal transplant (which had provided outstanding voice and swallowing function for more than a decade but was rendered nonfunctional by slowly progressive chronic rejection, necessitating removal at 14 years after transplant), P16 positive squamous cell carcinoma of the recipient's native tonsillar mucosa was discovered [2]. Although the tumor was successfully removed, this highlights the neoplastic risk associated from over a decade of high dose immunosuppression.

These serious complications raise a question about the ethics of performing VCA when more conservative approaches, unassociated with risk to life, exist although they often diminish the quality of life. One paper describes the public perception of hand and face transplants as procedures of a partly medical and partly elective nature, where people are less likely to wish to donate such tissues or organs compared to other organs and are more likely to believe recipients should make a financial contribution to receive these procedures [8]. However, another paper documents that the majority (75%) of patients that have undergone laryngectomy would be willing to receive a larynx transplant if offered. Notably, the first laryngeal transplant recipient who rejected the laryngeal transplant after 14 years expressed a desire to receive another transplant, despite the finding of a malignant complication [2, 9]. In an effort to decrease the ethical concerns, diminish the toxic side effects, and expand the indications for VCA use, a means to improve graft survival while reducing the doses of immunosuppressive drugs is required.

Administration of mesenchymal stem cells (MSCs) has emerged as a novel immunosuppressive therapy that allows the use of immunosuppressive drugs at lower doses or as monotherapies, or even their elimination. The “Pittsburgh Protocol,” utilized in five hand transplant patients, is the first attempt at cell therapy in clinical VCA, where whole donor bone marrow (BM) is intravenously infused following an inductive drug treatment to enhance the efficacy of tacrolimus monotherapy [10]. Although BM-derived MSCs (BMSCs) constitute only a 1/2500 fraction of BM, they appear to play a critical role in BM infusion immunomodulation [10]; therefore, great interest developed regarding the immunomodulatory properties of MSCs. MSCs can also be derived from adipose tissue after enzymatic digestion and culturing of the isolated stromal vascular fraction (SVF) to obtain the adipose-derived MSCs (ASCs) [11]. This review will analyze contemporary in vitro studies investigating the immunomodulatory potential and mechanisms of BMSCs and ASCs. It will also review the in vivo studies assessing their role in providing long-term tolerance to VCA.

## 2. Immunomodulatory Potential

Basic research of the immunomodulatory potential of BMSCs and ASCs suggests that culture conditions, tissue source, and donor characteristics may each have a significant impact on MSC characteristics. MSCs are repeatedly cultured (passaged) to increase their count. Increasing number of passages may lead to progressive cellular senescence, slowing of the proliferation rate, and, most importantly, a tendency to lose their immunomodulatory properties with passage numbers beyond seven [12, 13]. In vitro expansion of MSCs is usually achieved with *α*-minimal essential or Dulbecco's modified Eagle's media supplemented with 10% fetal bovine serum (FBS). New media enriched with human platelet lysate, autologous serum, or chemically defined serum-free media supplemented with human recombinant growth factors are in development to assure safety. However, it is unknown whether the various media affect the immunomodulatory properties of MSCs; thus, an investigation is warranted. Upon culture completion, it is possible to freeze and cryopreserve ASCs with thawed cells retaining the potential to grow, differentiate, and immunomodulate years after culture [14–16]. This creates the potential for a bank of third-party off-the-shelf cultured and cryopreserved ASCs available upon request. Recent investigations have determined that not all adipose tissue provides ASCs of equal quality, with adipose from aged donors being inferior, obesity accelerating aging, and variations in properties existing between anatomic locations [17–20]. Interestingly, MSCs do not constitutively exert their immunomodulating properties but have to be “primed” by inflammatory mediators released from activated immune cells, such as IFN-*γ*, IL1*β*, and TNF-*α* [21, 22]. Thus, it appears that tissue source, age, body mass index, and inflammatory status need to be taken into consideration for donor selection for allogeneic cell therapy or for assessing a need for priming of MSCs by inflammatory cytokines.

## 3. Immunomodulatory Mechanism

MSCs were originally isolated from bone marrow in the 1970s [40]. Extensive characterization of BMSCs has demonstrated that these cells are capable of suppressing MHC-mismatched lymphocytes and inhibiting activated T-cells, B-cells, NK cells, and dendritic cells [41–48]. Unfortunately, isolating BMSCs is an invasive process and yields relatively low numbers of MSCs [49]. Recently, MSCs derived from adipose tissue have been a focus of similar research due to their accessibility, abundance, and similar immunomodulatory capabilities [50]. Several studies have demonstrated that ASCs are comparable to BMSCs in their ability to modulate immune responses. In the early 2000s, Hicok et al. injected human ASCs into immunotolerant mice subjected to hind-limb ischemia demonstrating improved neovascularization and osteoid matrix formation, mounting an interest in ASC reparative properties [51]. In 2005, Puissant et al. pioneered investigation into the immunosuppressive capabilities of ASCs. ASCs inhibited PHA-stimulated peripheral blood mononuclear cell (PBMC) proliferation by 90%, utilizing both soluble mediators and cell contact to inhibit lymphocyte proliferation [52]. Yañez et al. investigated these soluble mediators by evaluating the effect ASCs had on secretory profiles of PHA-stimulated T-cells and T-cells stimulated by allogeneic cells, finding that TNF-*α*, IFN-*γ*, and IL-12 were significantly reduced [53]. This suggested the reduction in proinflammatory cytokines as the mechanism ASCs use to inhibit PBMC proliferation. Additionally, these authors show that allogeneic ASCs intravenously administered to mice experimentally subjected to graft-versus-host disease (GVHD) resulted in significant survival improvement [53]. Kang et al. investigated the soluble factors responsible for reducing proinflammatory cytokine production, discovering that ASCs increased mRNA expression of immunosuppressive mediators (TGF-*β*, HGF, IDO, and COX-2) when cocultured with activated leukocytes in vitro [54].

In addition to immunosuppressive cytokine production, Yousefi et al. discovered that murine ASCs significantly improved the clinical course of experimental autoimmune encephalomyelitis by increasing the production of CD4^+^CD25^+^FOXP3^+^ T-regulatory cells (T_regs_) [55]. Najar et al. reported that human ASCs inhibited proliferation of activated CD4^+^ and CD8^+^ T-cells in vitro [56]. They also demonstrated that PGE2 expression significantly increases in ASCs cocultured with activated lymphocytes and that inhibition of ASC PGE2 synthesis prevents MSC-mediated immunosuppression, suggesting an important role of PGE2 in the immunosuppressive capabilities of ASCs [56]. While most studies have focused on ASC modulation of T-cell proliferation, ASCs also inhibit plasma cell production of immunoglobulin, suppress plasma cell differentiation, and increase IL-10 producing B-regulatory cells [57].

ASCs and BMSCs share many similarities, but some significant differences exist. Morphologically, ASCs are significantly smaller than BMSCs, being only a third of the diameter when both are trypsinized [58]. This is significant since in vivo studies have demonstrated that MSCs are trapped in pulmonary capillary beds and liver sinusoids, and it is postulated that smaller cells may more effectively home to allografts [58]. In a clinical trial monitoring the efficacy of MSC therapy in the treatment of ischemic cardiomyopathy, pulmonary pooling led to transient pulmonary edema and worsening of cardiac function [59]. Within these distant capillary beds, MSCs had formed microemboli with transient anti-inflammatory paracrine function followed by apoptosis and necrosis [60]. Melief et al. quantitatively compared the immunosuppressive capabilities of ASCs to BMSCs, discovering that ASCs are more potent immunomodulators, since approximately threefold more BMSCs were necessary to obtain the same immunosuppressive effect on PBMC proliferation compared to ASCs, ASCs were more effective in inhibiting dendritic cell activation, and ASCs produced higher concentrations of anti-inflammatory cytokines when incubated with activated lymphocyte cultures [61]. Plock et al. further evidenced the in vitro superiority of ASCs, with near complete abolishment of T-cell responses compared to BMSCs which showed dose-dependent suppression [36]. Collectively, these in vitro data demonstrate that ASCs have the potential to modulate the innate and adaptive immune systems to a favorable environment for allotransplantation.

## 4. Preclinical In Vivo Studies of the Immunosuppressive Effect of MSCs in VCA

Table 1 includes all studies known to the authors that have assessed outcomes of MSC immunomodulatory therapy in the setting of animal VCA or skin allotransplantation [23–31, 33–37, 62]. Since skin is the most antigenic component of VCA and it has been reported to be the first tissue to show signs of rejection in human hand transplantations, skin allograft trials are a useful proxy for outcomes in a VCA setting [31, 32, 63]. Eleven of the sixteen studies listed in the table compared the allotransplant survival in the group treated with MSC alone versus control groups that received no MSC. Of these studies, ten reported improved graft survival, which was reported to be of statistical significance in eight of them. The remaining one study reported statistically significant shortening of the allogenic skin graft survival in rats following BMSC administration [26]. Nine of the studies assessed the long-term allotransplant survival in combined MSC and pharmacological immunosuppression regimens counted from the first day of discontinuing the pharmacological treatment. One rat developed long-term tolerance to its hind-limb allotransplant after having received antilymphocyte serum (ALS) induction in addition to early cyclosporine (CsA) treatment [28], and two did so following total body irradiation (TBI), antilymphocyte globulin (ALG), and CsA [34]; all other animals without MSC therapy suffered transplant rejection [26–30, 34–36]. Aksu et al. have been excluded from this analysis, since their regimen of bone marrow infusion caused GVHD almost unanimously in animals not treated with MSCs, so a clinically meaningful comparison cannot be made [24]. Of note, GVHD in rats treated with MSCs was greatly ameliorated. This phenomenon was also reported in a swine hind-limb allotransplant model [27]. These data demonstrate that MSC therapy as an adjunct to various regimens of conventional immunosuppression contributes to achieving an immunogenic tolerance to VCA in vivo.

## 5. Monitoring of Rejection and Local Immunomodulation 

Histologic monitoring of transplant rejection was undertaken in all included studies, most using Banff criteria (Table 2) [39]. Rejection severity was tissue and treatment group specific. The greatest degree of rejection was found in the dermal-epidermal junction, followed by the lymphoid tissue, and least in muscle [30]. Severe (grade 3) rejection was ubiquitous in the dermis of control allotransplants by postoperative day (POD) 14 and regularly present at POD 7. Animals treated with MSCs alone demonstrated improved early tolerance of the allotransplant histologically; however, at POD 14, dermal rejection was moderate (grade 2/3). In studies with pharmacologic immunosuppressant cohorts, all animals currently receiving treatment (with or without concurrent MSC therapy) showed no or minimal rejection (grade 0/1) in the dermis and muscle. Once pharmacologic immunosuppression was ceased, animals excluded from MSC treatment progressively demonstrated histologic rejection, but the rate of rejection varied. Kuo et al. found 3 weeks after CsA discontinuation in swine hemifacial allotransplant that grade 2/3 rejection was seen in the dermis and lymphoid tissue, whereas the same authors found minimal rejection in rat hind-limb VCA after an equal period of treatment-free follow-up [29, 30]. In animals treated both pharmacologically and with MSCs, microscopic rejection was significantly reduced in the treatment-free period and grade 0 or 1 rejection was seen in all tissues 3 weeks after treatment cessation in all animals. Grade 0 or 1 rejection was found in surviving allotransplants at study end points in all apposite trials [26–30, 34–36]. These data provide additional evidence that immunogenic tolerance can be stimulated by MSC therapy combined with pharmacologic induction. The potential for MSCs to home to allotransplant tissue appears to be vital for MSCs to induce allotransplant tolerance [58]. Two of our included animal studies assessed MSC homing with tissue biopsy on PODs 3 and 10, both finding significant populations of labeled MSCs in the subcutaneous layer of donor and recipient skin [27, 32].

In a small number of studies, additional immunomodulation monitoring was performed by in vivo quantification of circulating cytokine proteins in serum or gene levels of transcription factors regulating cytokine expression within tissues (Table 3) [25, 29–32]. However, the differences in the timing of analyses and the treatment regimens between groups in these studies pose a challenge to accurate interpretation of the paracrine mechanisms involved. Conditioned medium from cultured ASCs has been equally efficient to ASCs in improving allograft survival; furthermore, its effect on peripheral blood cytokines was greater with significantly reduced levels of IL-10, TNF-*α*, and IFN-*γ* [32], supporting the notion that the paracrine activity of MSCs may contribute to their immunomodulatory effects. The precise in vivo mechanisms still need further clarification.

Likewise, quantification of the populations of T_regs_ in allotransplant tissue and the circulation (Table 3) shows increased T_reg_ levels in allotransplant tissue, typically in greater number at the dermal-epidermal junction, in all studies that used this approach [27–30, 33] except one [31]. Comparing T_reg_ levels in the serum of animals treated with combined MSC and pharmacological immunosuppression in those with VCA tolerance versus those with VCA rejection shows a significantly greater number of peripheral T_regs_ in immunotolerant animals [34, 35]. One study reports a greater T_reg_ number in animals treated with ASC than with BMSC, each combined with pharmacological immunosuppression [36].

These data support the assertion that MSCs, in combination with pharmacological therapy, contribute to long-term VCA tolerance by both increasing the population of circulating T_regs_ and homing to allotransplant tissue, where they release chemokines that further recruit T_regs_ to sustain their abundance.

## 6. Preclinical In Vivo Evidence: Areas of Controversy

Sbano et al. are not alone in reporting worsened outcome with MSC therapy [25]. Inoue et al. reported significantly shortened cardiac allograft survival in rats that received combined MSC and CsA therapy compared to rats receiving CsA alone [64]. There is growing awareness that MSCs possess soluble factor secretion plasticity and their interaction with the local and systemic environment critically determines their paracrine functions; therefore, before MSCs can become a licensed therapy for clinical practice, further investigation into the culture, expansion, and priming of MSCs is warranted [65, 66]. This plasticity may explain variation in response between animals and studies. Likewise, the in vitro superiority of ASCs over BMSCs has not been borne out in in vivo studies combining MSC with ALS and tacrolimus exposure [36]. It is also possible that, compared to BMSCs, ASCs may have increased susceptibility to toxicity from select pharmacologic agents, with dose-dependent inhibition of their proliferation and immunosuppressive properties [36, 58]. Thus, the interactions between MSCs and pharmacological drugs require further investigation.

Particularly relevant to this review is the poorer survival of facial transplants compared to hind-limb transplants. This may highlight the challenge posed by the antigenic nature of facial transplants due to their high proportion of skin and lymphoid tissue or may be the result of the lack of vascularized BM within the transplant. Ramirez et al. compared VCA with and without BM and with BM in higher or lower proportion to skin by comparing among hind-limb transplants (containing bone and vascularized BM), myocutaneous hemiabdominal wall transplants, and transplants combining the two [35]. They found significantly longer survival in grafts including BM and where it was in higher proportion (hind-limb only). There were a significantly higher number of donor/recipient chimeric cells in the group with abundant BM, although chimerism was still present in recipients of allotransplants without BM. Furthermore, BM produced higher levels of chimeric cells compared to the chimerism induced by BMSCs or in vivo generated donor/recipient chimeric cells when cotransplanted with MCH-mismatched hemiface in a rat model [38]. These data support the hypothesis that long-term MSC-induced tolerance requires MSC immunomodulation in combination with sustained mixed chimerism and that BM may only support a limited mass of antigenic material. Concurrent ASC and BM infusion has been shown to increase mixed chimerism and skin allograft survival, although the long-term effects are uncertain [58]. Further research regarding MSC origin, dose, and timing is necessary.

Casiraghi et al. encountered a time-dose relationship where pretransplant MSC induction promoted murine renal allotransplant survival while posttransplant MSC therapy caused neutrophilic infiltration and rejection of the allotransplant [67]. This dichotomy is not seen in VCA. For example, Plock et al. found that single postoperative administration of MSCs provided successful immunomodulation and that outcomes were not improved by increasing MSC dose in their model [36]. It is postulated that syngeneic MSCs have a longer half-life in vivo than allogeneic MSCs; thus, single infusion may be more likely to be successful with their use [34]. Further research regarding optimal MSC dose and timing is necessary.

## 7. Clinical Evidence of MSC Safety and Efficacy

Concerns have been raised regarding the potential for MSC therapy to introduce cells that have undergone malignant transformation resulting in a malignancy in the recipient. These concerns were raised by preclinical trials showing an increased tumor burden in recipients; however, a recent systematic review investigating the safety of clinical MSC therapy found no association between MSC therapy and de novo malignancy, an increased risk of infection, nor any other serious complications [68, 69]. The single significant association was for transient fever following administration with no reports of acute infusion toxicity [69]. This evidence from a cohort of over 1000 patients supports the assertion of the “immunoprivilege” of MSCs and should allay fears over the safety of MSC therapy.

To date, MSCs have only been used as an induction therapy to induce immunotolerance in recipients of renal transplants in several small clinical trials and two large trials [70–72]. The randomized controlled trial of Tan et al. [71] compared 3 treatment groups in a cohort of 159 patients using autologous BMSCs: (1) Group A received standard-dose calcineurin inhibitors (CNI) plus BMSCs; (2) Group B received low-dose CNI plus BMSCs; and (3) Group C received standard-dose CNI plus anti-IL-2 receptor antibodies. Follow-up was one year. The study found that the BMSC groups had improved kidney function during the first month and were associated with fewer episodes of acute rejection and that these episodes were milder and more likely respondent to steroids after 6 (but not 12) months. They also had fewer adverse effects; that is, Group B suffered fewer infections. Patient and graft survival after 1 year was similar among all groups.

Vanikar and Trivedi undertook a prospective open-labeled 4-armed clinical trial with 916 patients [72]. Patients opting for standard therapy receiving triple immunosuppression of CNI, MMF, and prednisone were the controls, while those opting for tolerance induction protocol received a set of treatments including autologous ASC infusions in addition to standard therapy. Each group was subdivided into those with ≥1 haplomatch and those with poorly matched kidneys. Follow-up was four years. Baseline characteristics were similar between cohorts. The overall survival and mortality-censored graft survival at 1 and 4 years were similar among all patients with highly matched kidneys. But the ASC infusion had a remarkable impact on the immunosuppressive regimen at 4 years of follow-up. All patients in the control group were receiving triple immunosuppressant therapy compared to only 17% of patients in the ASC group. Furthermore, 78% of the latter were able to sustain good graft function in the absence of rejection on low-dose two-drug immunosuppressive therapy, and 5% of patients could be maintained on prednisone alone. In patients receiving poorly matched kidneys, the beneficial effects of ASC induction therapy were far more apparent: 89% versus 83% (controls) survival and 95% versus 75% (controls) mortality-censored graft survival at 4 years; better kidney function at 4 years; and lower incidence of severe acute rejection and chronic rejection. There was a similar trend in regard to immunosuppressive therapy with 60% of patients in the MSC group successfully maintained on two-drug therapy. Although biased by a lack of blinding, these figures provide significant evidence that ASCs can allow immunosuppressive dosage reduction in the clinical setting. The preferential positive effects of ASCs to those with poorly matched allotransplants imply that ASCs have a more potent immunomodulatory effect in the setting of highly immunogenic tissue transplant and that MSC induction therapy promotes long-term tolerance and improved function of human organ transplant.

## 8. Conclusion

VCA is a rapidly evolving realm of surgery with tremendous potential to restore form and function. A crucial restrictor of VCA clinical implementation is the extreme immunosuppressive regimen required, which is associated with a plethora of dangerous sequelae. Immunosuppression minimization or allotransplant tolerance could revolutionize allotransplantation within the head and neck and widen VCA indications to include defects after resection of malignant disease or congenital anomalies [73].

Proof of principle has been established for MSC immunomodulation and clinical trials support evidence from preclinical trials that induction therapy with MSCs (especially ASCs) can induce long-term tolerance to a highly immunogenic allotransplant in vivo, improve graft survival and function, and reduce immunosuppressive doses (thus reducing complication risk and cost of therapy) [74]. Furthermore, MSCs may improve VCA function beyond graft tolerance through improved neural regeneration. Further investigation of in vivo evidence of improved functional outcomes is awaited [75].

It is important that surgeons are aware of the potential for MSC induction therapy to overcome many of the obstacles to widespread VCA in clinical practice. More preclinical studies are needed before the promise of MSC-induced VCA tolerance becomes a clinical reality.

## Figures and Tables

**Table 1:** Preclinical in vivo studies assessing the immunomodulatory effect of MSC therapy in vascularized composite or skin allotransplantation.

Authors	Animal (number)	Allogeneic transplant	MSC type	MSC dose (days of infusion)	Other treatments (days of administration)	Days of therapy-free follow-up	Increased graft survival in MSC alone? (*P* value)	Percent of grafts surviving until end point
Bartholomew et al. (2002) [23]	Baboon (8)	Skin	BMSC allogeneic	20 × 10^6^ cells/kg (0, 3)	None		Yes (unstated)	

Aksu et al. (2008) [24]	Rat (44)	Skin	BMSC allogeneic	2-3 × 10^6^ cells (0, 7, 14, 21)	TBI (1), CsA (0–20), BM IV (0, 7, 14, 28)			

Sbano et al. (2008) [25]	Rat (62)	Skin	BMSC allogeneic	5.7 × 10^6^ cells/kg (0) 10.3 × 10^6^ cells/kg (0)	CsA (0–30)		No (<0.001)	

Pan et al. (2010) [26]	Rat (47)	Hind-limb	BMSC allogeneic	200 × 10^6^ cells (−30)	TBI (−30), RAPA (−30–100), ALG (−6, −4, −2), BMT (−30)	0^†^	Yes (unstated)	100

Kuo et al. (2009) [27]	Swine (23)	Hind-limb	BMSC allogeneic	1 × 10^7^ cells (−1, 3, 7, 14, 21)	TBI (−1), CsA (0–28)	280	Yes (0.02)	60

Kuo et al. (2011) [28]	Swine (16)	Hind-limb	BMSC allogeneic	1 × 10^7^ cells (−1, 1, 3, 7, 14, 21)	TBI (−1), CsA (0–28)	100	Yes (0.018)	67

Kuo et al. (2011) [29]	Rat (28–36)	Hind-limb	ASC allogeneic	2 × 10^6^ cells (7, 14, 21)	ALS (−4, 1), CsA (0–28)	180		89

Kuo et al. (2012) [30]	Swine (16)	Facial	BMSC allogeneic	2.5 × 10^7^ cells (−1, 1, 3, 7, 14, 21)	CsA (0–28)	67^††^	Yes (0.123)	0

Larocca et al. (2013) [31]	Mouse (21)	Skin	ASC allogeneic	5 × 10^5^ cells (1)	BM IV		Yes^§^ (<0.05)	

Lee et al. (2014) [32]	Mouse (48)	Skin	ASC or CM human	1 × 10^6^ cells or 0.3 mL CM^*∗*^ (0)	None		Yes^§§^ (<0.005)	

Jeong et al. (2014) [33]	Rat (21)	Hind-limb	BMSC allogeneic	1 × 10^5^ cells (0, 1, 2, 3)	None		Yes (<0.001)	

Cheng et al. (2014) [34]	Rat (32)	Hind-limb	ASC syngeneic	2 × 10^6^ cells (1)	TBI (−1), ALG (−1, 10), CsA (0–10)	140		67

Ramirez et al. (2014) [35]	Rat (47)	Hind-limb and/or HAW	ASC syngeneic	2 × 10^6^ cells (1, 8, 15)	ALS (−1, 10), CsA (0–10)	140		38 HLOMC, 0 HAW, 0 HLOMC + HAW

Plock et al. (2015) [36]	Rat (35)	Hind-limb	BMSC or ASC allogeneic	1 × 10^6^ or 5 × 10^6^ cells (1)	ALS (−4, 1), tacrolimus (0–20)	100		46 BMSC, 47 ASC

Davis et al. (2014) [37]	Mouse (38)	Skin	BMSC or ASC human	5 × 10^5^, 3 × 10^6^ cells, respectively (7)	Anti-CD4/CD8 mAbs (0, 2, 5, 7, 14), busulfan (5)		Yes (unstated)	

Hivelin et al. (2016) [38]	Rat (24)	Hemifacial	BM, BMSC, D/RCC	100 × 10^6^, 5 × 10^6^, 10 × 10^6^ cells, respectively; intraosseous injections	Anti-*αβ*-TCR mAb, CsA (1–7)	Control: 25–29^††^, BMSC: 27–32^††^	Yes (0.03)	0

↑: increased; ALG: antilymphocyte globulin; ALS: antilymphocyte serum; ASCs: adipose tissue-derived mesenchymal stem cells; BM: bone marrow; BMSC: bone marrow-derived mesenchymal stem cell; BMT: bone marrow transplant; CM: conditioned media; CsA: cyclosporine A; D/RCC: donor-recipient chimeric cells; HAW: hemiabdominal wall; mAbs: monoclonal antibodies; MSC: mesenchymal stem cell; RAPA: rapamycin; TBI: total body irradiation; HLOMC: hind-limb osteomyocutaneous combined flap. ^*∗*^CM from equal number of cells and concentrated 25-fold; ^†^100-day follow-up on CsA monotherapy; ^††^when final surviving graft perished; ^§^only for donor-specific ASC; ^§§^for both cells and CM.

**Table 2:** The Banff 2007 working classification of skin-containing composite tissue allograft pathology [39].

Banff Grade	Rejection severity	Histopathological features
0	No rejection	No or rare inflammatory infiltrates
1	Mild	Mild perivascular infiltration and no involvement of the overlying epidermis
2	Moderate	Moderate-to-severe perivascular inflammation with or without mild epidermal and/or adnexal involvement
		No epidermal dyskeratosis or apoptosis
3	Severe	Dense inflammation and epidermal involvement with epithelial apoptosis, dyskeratosis, and/or keratinolysis
4	Necrotizing acute rejection	Frank necrosis of epidermis or other skin structures

**Table 3:** Monitoring of immunomodulation in preclinical in vivo studies.

Authors	IL-6	TGF-*β*1	IL-10	TNF-*α*	IFN-*γ*	*T*_regs_
Bartholomew et al. (2002) [23]	—	—	—	—	—	—

Aksu et al. (2008) [24]	—	—	—	—	—	—

Sbano et al. (2008) [25]	—	Skin mRNA MSC only: ND MSC + CsA: *P* < 0.01	Skin mRNA MSC only: ND MSC + CsA: ND	Skin mRNA MSC only: ND MSC + CsA: *P* < 0.03	Skin mRNA MSC only: *P* < 0.01 MSC + CsA: ND	—

Pan et al. (2010) [26]	—	—	—	—	—	—

Kuo et al. (2009) [27]	—	—	—	—	—	Circulation and skin MSC + CsA: ↑ *P* < 0.05 MSC only: ND

Kuo et al. (2011) [28]	—	—	—	—	—	Circulation and skin MSC + CsA: ↑ *P* < 0.05 MSC only: ND

Kuo et al. (2011) [29]	—	Circulation MSC + CsA: ↑ *P* < 0.05	Circulation MSC + CsA: ↑ *P* < 0.05	—	—	Circulation and skin MSC + CsA: ↑ *P* < 0.05

Kuo et al. (2012) [30]	Skin: ↓ *P* < 0.05	Skin MSC + CsA: ↑ *P* < 0.05 MSC only: ↑ *P* < 0.05 Circulation: ND	Circulation MSC + CsA: ↑ *P* < 0.05 MSC only: ↑ *P* < 0.05	Circulation MSC + CsA: ↓ *P* < 0.05 MSC only: ↓ *P* < 0.05	Circulation: ND	Circulation and skin MSC + CsA: ↑ *P* < 0.02 MSC only: ND

Larocca et al. (2013) [31]	Lymph node: ↑ *P* < 0.05 Skin: ND	Lymph node: ND Skin: ND	Lymph node: ↑ *P* < 0.05 Skin: ND	—	Lymph node: ↑ *P* < 0.05 Skin: ND	Lymph node: ↑ *P* < 0.05 Skin: ND

Lee et al. (2014) [32]	Circulation ASC: ↓ *P* < 0.05 CM: ↓ *P* < 0.05	—	Skin mRNA ASC: ND CM: ↓ *P* < 0.05	Skin mRNA ASC: ND CM: ↓ *P* < 0.05	Skin mRNA ASC: ND CM: ↓ *P* < 0.05	—

Jeong et al. (2014) [33]	—	—	—	—	—	Skin and muscle: ↑ *P* < 0.05

Cheng et al. (2014) [34]	—	—	—	—	—	Circulation VCA tolerant: ↑ *P* < 0.05 VCA intolerant: ND

Ramirez et al. (2014) [35]	—	—	—			Circulation VCA tolerant: ↑ *P* < 0.05 VCA intolerant: ND

Davis et al. (2014) [37]	—	—	—	—	—	Circulation and splenocytes: ↑ *P* < 0.05

Plock et al. (2015) [36]	—	—	—	—	—	Circulation ASC: ↑ *P* < 0.01 BMSC: ND

ND: not different statistically; ↑: increased; ↓: decrease.

## Abbreviations

ALS: Antilymphocyte serum
ASCs: Adipose-derived mesenchymal stem cells
BM: Bone marrow
BMSCs: Bone marrow-derived mesenchymal stem cells
CNI: Calcineurin inhibitors
CsA: Cyclosporine
GVHD: Graft-versus-host disease
IFN-*γ*: Interferon gamma
IL: Interleukin
MSCs: Mesenchymal stem cells
PBMC: Peripheral blood mononuclear cell
PGE2: Prostaglandin E2
POD: Postoperative day
TNF-*α*: Tumor necrosis factor-alpha
T_regs_: T-regulatory cells
VCA: Vascularized composite allotransplant.
